# The COMBREX Project: Design, Methodology, and Initial Results

**DOI:** 10.1371/journal.pbio.1001638

**Published:** 2013-08-27

**Authors:** Brian P. Anton, Yi-Chien Chang, Peter Brown, Han-Pil Choi, Lina L. Faller, Jyotsna Guleria, Zhenjun Hu, Niels Klitgord, Ami Levy-Moonshine, Almaz Maksad, Varun Mazumdar, Mark McGettrick, Lais Osmani, Revonda Pokrzywa, John Rachlin, Rajeswari Swaminathan, Benjamin Allen, Genevieve Housman, Caitlin Monahan, Krista Rochussen, Kevin Tao, Ashok S. Bhagwat, Steven E. Brenner, Linda Columbus, Valérie de Crécy-Lagard, Donald Ferguson, Alexey Fomenkov, Giovanni Gadda, Richard D. Morgan, Andrei L. Osterman, Dmitry A. Rodionov, Irina A. Rodionova, Kenneth E. Rudd, Dieter Söll, James Spain, Shuang-yong Xu, Alex Bateman, Robert M. Blumenthal, J. Martin Bollinger, Woo-Suk Chang, Manuel Ferrer, Iddo Friedberg, Michael Y. Galperin, Julien Gobeill, Daniel Haft, John Hunt, Peter Karp, William Klimke, Carsten Krebs, Dana Macelis, Ramana Madupu, Maria J. Martin, Jeffrey H. Miller, Claire O'Donovan, Bernhard Palsson, Patrick Ruch, Aaron Setterdahl, Granger Sutton, John Tate, Alexander Yakunin, Dmitri Tchigvintsev, Germán Plata, Jie Hu, Russell Greiner, David Horn, Kimmen Sjölander, Steven L. Salzberg, Dennis Vitkup, Stanley Letovsky, Daniel Segrè, Charles DeLisi, Richard J. Roberts, Martin Steffen, Simon Kasif

**Affiliations:** 1 New England Biolabs, Ipswich, Massachusetts, United States of America; 2 Bioinformatics Program, Boston University, Boston, Massachusetts, United States of America; 3 Department of Biomedical Engineering, Boston University, Boston, Massachusetts, United States of America; 4 Diatom Software LLC, Holliston, Massachusetts, United States of America; 5 Program for Evolutionary Dynamics, Harvard University, Cambridge, Massachusetts, United States of America; 6 Department of Mathematics, Emmanuel College, Boston, Massachusetts, United States of America; 7 Department of Chemistry, Wayne State University, Detroit, Michigan, United States of America; 8 Department of Plant and Microbial Biology, University of California, Berkeley, California, United States of America; 9 Department of Chemistry, University of Virginia, Charlottesville, Virginia, United States of America; 10 Department of Microbiology and Cell Science, University of Florida, Gainesville, Florida, United States of America; 11 Department of Microbiology, Miami University, Oxford, Ohio, United States of America; 12 Department of Chemistry, Georgia State University, Atlanta, Georgia, United States of America; 13 Bioinformatics and Systems Biology, Sanford Burnham Medical Research Institute, La Jolla, California, United States of America; 14 Department of Biochemistry and Molecular Biology, University of Miami, Miami, Florida, United States of America; 15 Department of Molecular Biophysics and Biochemistry, Yale University, New Haven, Connecticut, United States of America; 16 School of Civil and Environmental Engineering, Georgia Institute of Technology, Atlanta, Georgia, United States of America; 17 European Bioinformatics Institute, Wellcome Trust Genome Campus, Hinxton, Cambridgeshire, United Kingdom; 18 Department of Medical Microbiology and Immunology, and Program in Bioinformatics, University of Toledo, Toledo, Ohio, United States of America; 19 Department of Biochemistry and Molecular Biology, Pennsylvania State University, University Park, Pennsylvania, United States of America; 20 Department of Biology, University of Texas-Arlington, Arlington, Texas, United States of America; 21 Spanish National Research Council (CSIC), Institute of Catalysis, Madrid, Spain; 22 National Center for Biotechnology Information (NCBI), National Institutes of Health (NIH), Bethesda, Maryland, United States of America; 23 Department of Library and Information Sciences, University of Applied Sciences Western Switzerland, Geneva, Switzerland; 24 Bibliomics and Text Mining Group, Swiss Institute of Bioinformatics, Geneva, Switzerland; 25 J. Craig Venter Institute, Rockville, Maryland, United States of America; 26 Biological Sciences, Columbia University, New York, New York, United States of America; 27 Bioinformatics Research Group, Artificial Intelligence Center, SRI International, Menlo Park, California, United States of America; 28 Department of Microbiology, Immunology, and Molecular Genetics, University of California, Los Angeles, Los Angeles, California, United States of America; 29 Department of Bioengineering, University of California, San Diego, La Jolla, California, United States of America; 30 Department of Chemistry, Indiana University Southeast, New Albany, Indiana, United States of America; 31 Wellcome Trust Sanger Institute, Wellcome Trust Genome Campus, Hinxton, Cambridgeshire, United Kingdom; 32 Department of Chemical Engineering and Applied Chemistry, University of Toronto, Toronto, Ontario, Canada; 33 Center for Computational Biology and Bioinformatics, Columbia University, New York, New York, United States of America; 34 Integrated Program in Cellular, Molecular, Structural, and Genetic Studies, Columbia University, New York, New York, United States of America; 35 Department of Computing Science, University of Alberta, Edmonton, Alberta, Canada; 36 School of Physics and Astronomy, Tel Aviv University, Tel Aviv, Israel; 37 Berkeley Phylogenomics Group, University of California, Berkeley, California, United States of America; 38 Departments of Medicine and Biostatistics, McKusick-Nathans Institute of Genetic Medicine, Johns Hopkins University School of Medicine, Baltimore, Maryland, United States of America

## Abstract

Experimental data exists for only a vanishingly small fraction of sequenced microbial genes. This community page discusses the progress made by the COMBREX project to address this important issue using both computational and experimental resources.

## Introduction

Prior to the “genomic era,” when the acquisition of DNA sequence involved significant labor and expense, the sequencing of genes was strongly linked to the experimental characterization of their products. Sequencing at that time directly resulted from the need to understand an experimentally determined phenotype or biochemical activity. Now that DNA sequencing has become orders of magnitude faster and less expensive, focus has shifted to sequencing entire genomes. Since biochemistry and genetics have not, by and large, enjoyed the same improvement of scale, public sequence repositories now predominantly contain putative protein sequences for which there is no direct experimental evidence of function. Computational approaches attempt to leverage evidence associated with the ever-smaller fraction of experimentally analyzed proteins to predict function for these putative proteins. Maximizing our understanding of function over the universe of proteins *in toto* requires not only robust computational methods of inference but also a judicious allocation of experimental resources, focusing on proteins whose experimental characterization will maximize the number and accuracy of follow-on predictions.

COMBREX (COMputational BRidges to EXperiments, http://combrex.bu.edu) is an NIH-funded enterprise that has brought computational and experimental biologists together, with the goal of greatly improving our overall understanding of microbial protein function [1],[2]. Since its inception, it has made significant progress toward the following goals: identifying the minority of proteins that have already been experimentally characterized, serving as a public repository of novel protein function predictions made by diverse methods, producing a clear chain of evidence from experiment to prediction, identifying (“recommending”) those functional predictions whose verification will contribute most to our overall understanding of protein function, and actually funding the experiments to test function. The recommendation system is a proof of concept based on active learning principles and includes, for a given protein, criteria including phylogenetic distribution of its protein family, biological and clinical phenotypes associated with it, the availability of protein structure data, and its sequence distance from experimentally determined proteins or from the other proteins in its family.

COMBREX comprises several interrelated efforts. First, the project is building a community of researchers (the *COMBREX Community*) committed to achieving the goals above. Second, the project maintains a web-accessible database (the *COMBREX Database*) of known and predicted functions for microbial proteins. The database search features enable biologists to identify predictions whose experimental verification is particularly important. Finally, the project issues small monetary awards (*COMBREX grants*) to biologists to fund the experimental testing of such predictions. In this paper, we provide a brief review of COMBREX, focusing on its overall design, its computational resources, and the experimental results from the first phase of the project.

## Overview of COMBREX

**Figure 1:**
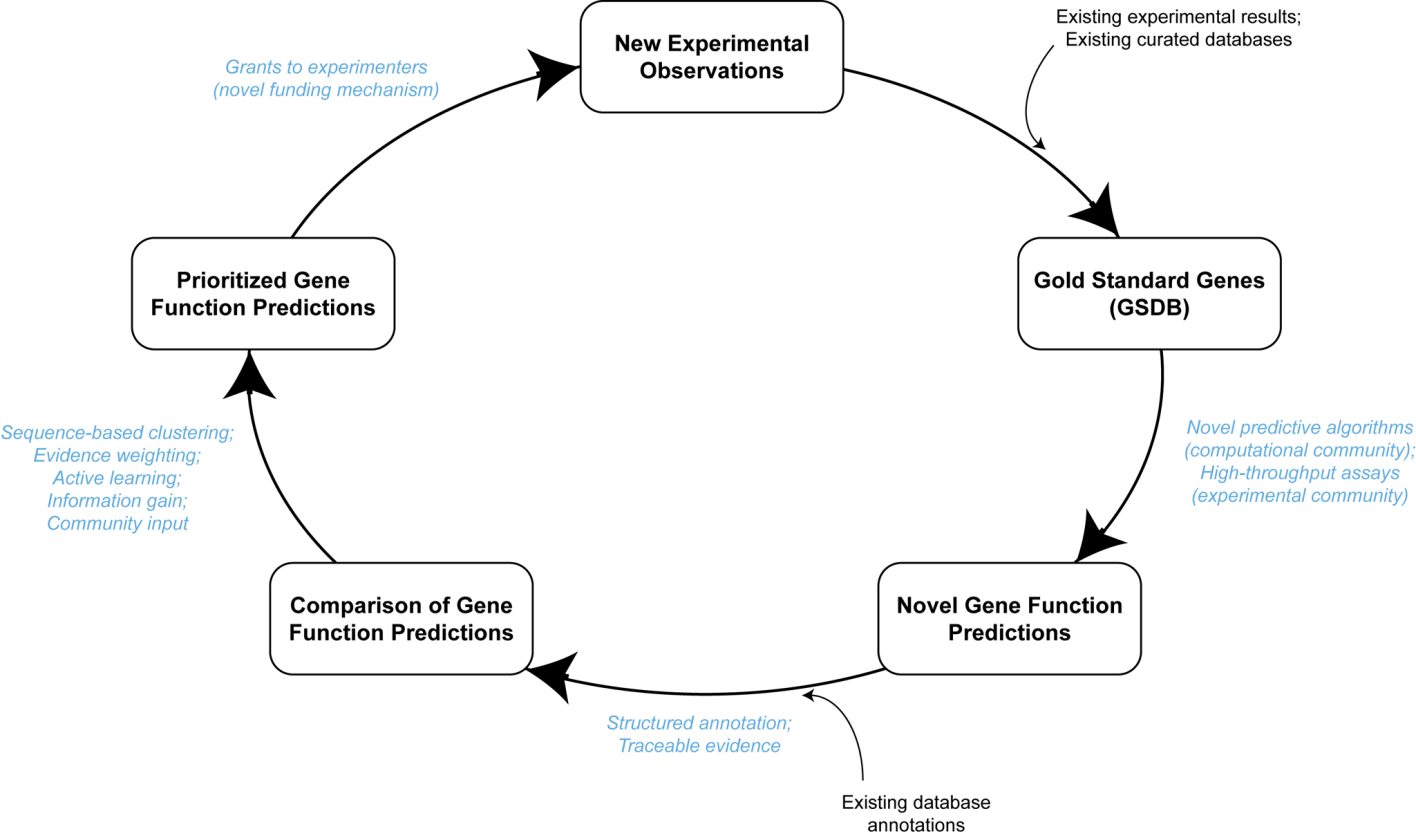
Schematic overview of the computational and experimental contributions of COMBREX and its users, and the interrelationships of these contributions. Data and results specific to COMBREX are shown in boxes. External data imported into COMBREX are also shown, with arrows indicating entry points into the cycle. Methodology employed by COMBREX and its users is shown in blue type, as it is used to generate data. Not shown are two critical contributions to COMBREX: genome and cluster data imported from NCBI RefSeq and ProtClustDB, respectively, and NIH funding, which enables the grants that COMBREX issues to experimental laboratories.

The activities of the COMBREX Community are summarized in Figure 1. As a starting point, we identify those proteins with experimentally confirmed functions (a functional “ground truth”). The COMBREX Community and its collaborators have assembled and are in the process of curating such a set, called the Gold Standard Database (GSDB). This set of known sequence-function relationships will ultimately serve as the basis for making predictions for similar proteins whose functions have not been experimentally determined and can be used to train other types of prediction-generating algorithms. Currently, the GSDB can be selectively accessed through the COMBREX Database by searching for proteins whose functions are experimentally determined.

**Figure 2:**
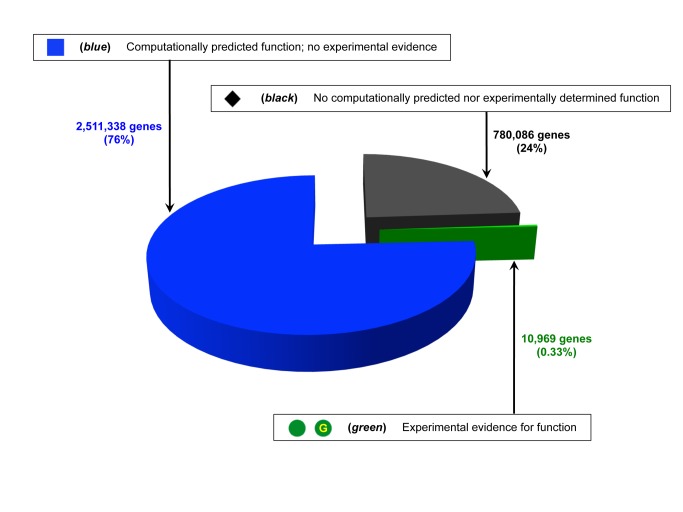
Definitions of COMBREX functional status symbols and fractions of microbial genes in COMBREX in each status category. Experimentally characterized proteins are *green*. (Those in the *green* set that have been manually curated by the GSDB are also marked with a gold “G.”) Proteins with functional predictions but no experimental evidence are *blue*. Proteins with no available functional predictions are *black*.

The objectives of the COMBREX Database are to act as a comprehensive repository of protein function predictions and experimental data, and to recommend important predictions to researchers for experimental analysis. Approximately 3.3 million proteins from more than 1,000 completely sequenced microbial genomes are represented in the database, and these are associated with about 2.5 million predictions of function. The *functional status* of each protein (that is, whether the function is known through direct observation, through prediction, or not at all) is summarized in Figure 2: experimentally characterized proteins are designated *green*, proteins with functional predictions *blue*, and those with no available predictions *black* (see Materials and Methods in Text S1 for further description of the color coding). The small fraction of experimentally characterized proteins is necessarily an underestimate because the GSDB is still a work in progress, but we estimate the true number is likely no more than ten-fold larger. The fraction of proteins with at least one computationally predicted function (76%) is by far the largest category, although the degree to which the prediction specifies a precise function varies widely.

Predictive models learn the most about a set of proteins through the experiment that produces the maximum gain of information over the entire set, and so identifying such experiments is of critical importance. Protein function predictions within the COMBREX Database are prioritized based on the expected information to be gained by their experimental testing. Information gained from experiments can be defined formally using probabilistic criteria [3], but can be understood intuitively in terms of the number of proteins for which predictions can be made, and the accuracy of those predictions, using the new experimental evidence. The recommendation system that performs the prioritization is intended to provide guidance to experimental researchers interested in applying for COMBREX grants. These grants are issued to biochemists and molecular biologists to enable the experiments needed to characterize specific microbial proteins, giving preference to those of “high priority” as identified by this system. The results of successful experiments can then be added to the GSDB, thereby completing the cycle depicted in Figure 1. COMBREX grants are dependent upon external funding, and the first round of awards was generously supported by the NIH using a novel funding mechanism. COMBREX continues to seek additional sources of funding to enlarge the participating community internationally.

## COMBREX Grants: Experiments Funded

Our funding model encourages the experimental characterization of proteins through small-scale funding of many laboratories using grants directly managed by COMBREX. Although high-throughput methods may ultimately allow for the study of many proteins simultaneously, at present the most effective way to accurately characterize protein function is through the dedicated examination of individual proteins. In order to maximize the value of COMBREX grants, they are preferentially issued to laboratories with demonstrated experience in the proposed assays. There are experimental and economic efficiencies to be gained by this in that these laboratories will typically already possess many of the reagents required for the assay, including relevant substrate libraries, as well as personnel with the expertise to conduct the assays rapidly. In addition, we advocate the testing of several members of a given protein family within a single grant whenever possible, since once all the necessary components are in place to test a single protein, there are only marginal increases in cost and labor needed to test multiple similar proteins. Importantly, should these similar proteins demonstrate different functions, this approach has the potential to delineate functional boundaries in sequence space, improving the follow-on predictions for many other proteins. All COMBREX grant applications are reviewed by an external panel of scientists to ensure that the proposed work is scientifically sound and that each laboratory is well suited to the proposed tasks.

In the first year of the project, COMBREX-funded and COMBREX-associated experimental efforts have initiated the examination of 140 proteins. (Funded teams can be found on the COMBREX website, at http://combrex.bu.edu/acknowledgments, and a complete list of these proteins can be found in Table S1.) In the ideal case that all of the proposed experiments are successful, the potential impact of these experiments in terms of follow-on predictions would be significant: the 140 proteins reside in Protein Clusters containing in total more than 3,200 proteins (resulting in high-confidence predictions for these) and are similar to over 60,000 proteins with BLAST *E*-values less than 1e-05 (resulting in lower-confidence predictions for these). Furthermore, among these 140 proteins are encoded eight Pfam-defined domains of unknown function (DUFs), resulting in novel predictive insights for all other proteins containing these DUFs (a total of 1,610 in the COMBREX Database). Finally, 37 of these 140 proteins contain a total of 28 unique Pfam-defined domains shared with human proteins, providing functional insights that may impact human health.

Research on about half of these proteins has been successfully completed, and results for some have been published [4]–[8], while research on the other half is still in progress. For those results that have been reported, 65% of proteins (44 of 68) have been verified to have the predicted function described in the COMBREX grant proposal, while no activity was observed for the remaining 24 (Table S1). Nine manuscripts funded by these COMBREX grants have been submitted for publication or are in preparation. As examples, we highlight the results of three COMBREX grants in Text S1.

## Connecting Function Predictions with Experimental Data

A major effort of COMBREX is to make predictions of gene function traceable to their experimental underpinnings. This knowledge is critical to any researcher attempting to assess the probability that a particular prediction is correct. Unfortunately, this information has generally not been maintained in most databases. Indeed, it is frequently unclear whether an annotated function describes the results of an experiment performed on that protein or is an inference made based upon homology to some other protein on which the experiment was performed.

In the COMBREX Database, this trace should ideally make clear the method used to generate the prediction, the input to the method, and the confidence in the <*gene*, *prediction*> pairing as measured by the method's scoring scheme. In practice, this is not always possible, particularly for unsourced annotations imported from public databases. However, direct comparison of such unsourced annotations with COMBREX-supported GSDB-based similarity assessment can provide some measure of confidence. For many uncharacterized proteins, COMBREX provides predictions based on sequence similarity to GSDB proteins: we note the GSDB protein that is the source of the prediction, the experimentally determined function, the publication(s) describing those experiments, and the degree of sequence similarity. As additional proofs of concept, we have explored similarities based upon protein structure and protein domain composition to assess the extent to which the experimental data in the GSDB can generate predictions for the remaining uncharacterized proteins. Specifically, we sought to determine the fractions of all uncharacterized proteins (*blue* or *black*) that can be related to experimentally characterized proteins (*green*) through sequence and domain-composition similarity under various thresholds. Results of these analyses, which are described in Text S1, show that existing experimental information can provide functional insight into more than half of all uncharacterized proteins.

## Prioritizing Predictions for Experimental Testing

Given the enormous mismatch in the rates of gene discovery by DNA sequencing and protein function confirmation by experiment, there is a compelling motivation to identify those proteins for which experimental results would be maximally informative in terms of follow-on predictive power. The COMBREX Database attempts to prioritize predictions based on the expected information to be gained by their experimental testing. We envision eventually employing a comprehensive metric based on probabilistic functional linkage [12]–[14] to assess “importance” using information theoretical principles. Our current recommendation system is a prototype that uses several relatively simple criteria to identify “important” proteins and make funding decisions.

The first criterion is the functional status of proteins assigned by COMBREX, with the rationale being to focus on those that have testable predicted functions but no associated experimental evidence (*blue* genes [Figure 2]). Second, when recommending proteins to examine from within a large family (cluster), COMBREX recommends candidates based on two properties: genome of origin and position within the cluster. We have chosen two “focus organisms,” *Escherichia coli* K-12 MG1655 and *Helicobacter pylori* 26695, for which we would like to obtain a large amount of experimental evidence, and we encourage confirming predictions from genes in these two strains. If a cluster does not contain a member from either of these genomes, COMBREX recommends gene(s) with the shortest average sequence distance to all other members of the cluster, in an attempt to select a gene most likely to be representative of the family (see Materials and Methods in Text S1). Third, we recommend proteins from larger protein families over those from smaller families, where a “family” is a super-cluster as defined by ProtClustDB (Materials and Methods in Text S1). Under the assumption that families are isofunctional, experimental evidence from one protein is likely to have an impact on our total understanding of protein function that is proportionate to the size of its family. This concept has been the subject of one published list of proposed experimental targets [15]. Finally, we recognize that there are significant contributory factors to “importance” that are independent of family size or sequence similarity. Examples of such factors might include being a key member of a particular biochemical pathway, having a biochemical function not previously identified experimentally, indicating functional diversification within a family previously thought to be isofunctional, or being associated with a phenotype of interest. This last factor benefits greatly from community input, and we encourage Community members to nominate proteins they believe to be important. A more detailed discussion of prioritization criteria can be found in Text S1.

Taking the above criteria into consideration, COMBREX has identified 100 genes we believe are of high priority for experimental analysis (http://combrex.bu.edu/top100) and specifically encourage proposals to characterize these proteins.

## Toward a Gold Standard Database

Experimental observations provide the foundation on which all functional predictions rest. In order to properly trace predictions to experiments, as well as to intelligently select maximally informative proteins for future experimental testing, one requires comprehensive knowledge of the identities of previously characterized proteins. In collaboration with NCBI, JCVI, and UniProt, we have begun assembling such a comprehensive set, namely the GSDB. A schema for the nomination and inclusion of genes in the GSDB is shown in Figure S4. First, candidate genes with functions that are believed to be experimentally determined are identified, either by importation from other curated databases such as EcoCyc, CharProtDB (JCVI) [16],[17], REBASE, and UniProtKB/Swiss-Prot, or by “nomination” by COMBREX Community members via the website. Once identified, candidate genes are manually examined by volunteer curators to see if they meet the criteria for inclusion in the GSDB.

Two criteria must be met for a gene/protein to meet the curation standards. First, the biochemical function of the gene product must have been determined experimentally in a published work or fully documented in a public database. Second, the DNA and/or protein sequence of the precise protein whose function was determined must also be known and be publicly available. Typically, this involves knowing with some precision the bacterial or archaeal strain from which the experimentally determined protein was isolated or cloned. These criteria are specified to ensure an unambiguous correspondence between sequence and function.

At present, the GSDB is small but growing; statistics are shown in Table 1. While the total number of experimentally characterized proteins is unknown, we estimate the number to be well above 50,000. The open-source, collaborative nature of COMBREX and partnering databases, combined with extensive participation from the scientific community at large, will be required for comprehensive identification of characterized proteins. We encourage everyone to nominate the proteins about which they have knowledge using the simple submission form at the COMBREX website (http://combrex.bu.edu/gold_form; requires registration) and to volunteer to help curate candidate GSDB proteins.

**Table 1:** Summary statistics for the GSDB.

GSDB Status	COMBREX Status	No. Genes
*Total records*		13,665^a^
Curated, accepted (GSDB)	*green* (marked with G)	164
Curated, rejected	*blue*	26
Not yet curated (GSDB queue)	*green*	13,475
**Source of Records**		
UniProt		4,017
REBASE		1,058
COMBREX		16
CharProtDB		8,574

a Of these records, 10,969 are currently represented in the COMBREX Database. The remaining records are primarily eukaryotic proteins.

## A Community-Based Model

COMBREX was initiated in response to a 2004 *PLOS Biology* editorial that proposed a community-wide effort to better understand the proteins encoded in the genomes we are continuing to sequence [1]. For success, the project relies on community participation for three major efforts: biochemical study of proteins by experimental biologists, computational function prediction by computational biologists, and manual curation of experimental information in the GSDB.

The biochemical effort by COMBREX is predicated on three principles: prioritization of experiments, parallelization of effort, and dissemination of results. Since we are limited to funding a relatively small number of experiments, prioritization is intended to guide us toward preferentially funding those experiments that can tell us the most not just about the specific proteins under study, but about other proteins for which these experimental results can generate predictions. The prioritization system, though rudimentary in its current form, is formally grounded in machine learning, specifically in active learning theory [3],[18],[19] (see Text S1).

Dissemination of the experimental results of COMBREX grants involves updating the GSDB, which leads directly to the generation of computational functional predictions for other proteins. The community of biologists relies heavily on gene and protein “annotations” in public databases for this predictive information, but these have several long-recognized shortcomings: the process by which a given annotation was generated is typically not transparent, the information is not always current with published literature, the error rate among these annotations can be high, and many lower-throughput methods of functional inference are not utilized [20]–[22]. Therefore, reliance on any one database for the predictive evidence COMBREX needs to effectively prioritize proteins would be unwise. We have therefore taken the approach not of selecting or generating the single best functional prediction for a given protein, but rather serving as a repository of predictions from many sources, which can be compared and evaluated using both statistical and biological criteria. While we work closely with several groups that specialize in benchmarking and competition, we also seek to identify methodologies that have complementary capabilities. This approach opens the door for the dissemination of results from specialized algorithms for functional prediction in a way not previously possible. The emphasis on function predictions, the documentation of evidence for these predictions, and the prioritization of uncharacterized proteins for experimental testing distinguish COMBREX from other publicly available microbial genomics resources such as IMG [23], SEED [24], GOLD [25], BioCyc [26], and others, each of which have their own unique emphasis.

The GSDB project, which requires the distillation of decades of published literature, also requires public participation through what we envision to be a crowdsourcing model. We have assembled preliminary data through collaboration with UniProt and with CharProtDB, a partially curated set of proteins with experimental evidence assembled by JCVI to serve as a source of evidence for its microbial genome annotation pipeline. However, manual curation or wiki-style collaboration will ultimately be needed to ensure the completeness of the information and the precise linkage of sequence and function. Our best hope for the success of the fledgling GSDB is broad participation from the experimental community in identifying characterized proteins and performing the necessary curation.

The public participation encouraged and required by COMBREX may have the additional benefit of exposing younger students to the biological sciences. The small-scale grant model that COMBREX has employed enables participation at the undergraduate level for appropriately equipped laboratories, since the necessary assays are frequently straightforward, self-contained in scope, and have technical challenges that can easily be met by beginning students with appropriate supervision. Furthermore, curricula built around teaching the techniques of cloning, protein purification, and biochemical assay to multiple students can be readily adapted to testing multiple related proteins in parallel. As an example of COMBREX-funded undergraduate participation, students in one laboratory section at the University of Virginia under the supervision of Linda Columbus were able to successfully investigate biochemical activities and enzyme kinetics for three previously uncharacterized proteins: TM0441 (results of different substrates further support the findings of Rodionova and colleagues [27] [and see Text S1]) and TM0542 from *Thermotoga maritima*, and Ta0880 from *Thermoplasma acidophilum* DSM1728 [28]. COMBREX hopes to continue collaboration with this group (http://biochemlab.org) and to replicate these successes as part of an educational component at numerous undergraduate institutions.

## Concluding Remarks

COMBREX is attempting to leverage relatively scant experimental resources to understand a large and growing collection of microbial proteins, the vast majority of which will likely never be directly functionally characterized. Computational predictions must continue to provide the basis for our understanding of most proteins. It is imperative that these predictions be as reliable as possible, and whenever possible, traceable to the experiments that provided the evidence for each prediction. When allocating experimental resources for this task, not all proteins are of equal benefit. In the most simplistic sense, characterization of a judiciously chosen protein generates or improves predictions for many other proteins across many genomes, while characterization of a protein related to few or no other proteins (often referred to as an ORFan [29],[30]) may have a much smaller impact. Despite the large number of genome sequences already available, new ORFans continue to appear at a significant frequency, leading some to estimate that the bacterial pan-genome may be of infinite size [31]. This suggests that a complete understanding of all bacterial proteins may be impossible, hence the need for prioritization. As an alternative to complete understanding, as proof of concept we adopted the twin goals of pushing our overall understanding toward the asymptote (by giving priority to conserved genes) and working toward the complete understanding of all proteins in one or a few genomes (by identifying “focus organisms”). With community participation on the experimental, computational, and curatorial sides, we feel these goals are within reach.

**Predictions of Protein Function**

Sources of predictions:

- COMBREX Community members, who can submit the results of their novel computational algorithms. We recognize that no single computational framework is likely to provide the most, or the best, predictions for all genes, and so we encourage the submission of predictions from a wide range of methodologies.
- Results of high-throughput experimentation, which may provide general clues to the functional role(s) of a protein.
- Functional associations generated by COMBREX's own algorithms, which link uncharacterized proteins to sufficiently similar GSDB proteins.
- Annotations available in publicly available databases such as the UniProt Knowledgebase (UniProtKB) [9], ProtClustDB [10], and RefSeq [11]. This group is by far the largest contributor, and COMBREX views any annotation or assignment of protein function that is not explicitly based on the experimental testing of that protein as a prediction.

Current progress: Currently, six computational teams have submitted function predictions to COMBREX, covering a total of 10,254 individual proteins (Table S5). We strongly encourage additional computational groups with published sets of predictions to consider submission to COMBREX, which can help publicize predictions and aid in the recruitment of experimentalists to test them. COMBREX does not have as a goal the comparison of the relative accuracies of various methods, but rather aims to integrate predictions from different sources and methodologies to gain the most complete possible picture of predicted functions for each protein. Users may browse and compare the predictions to draw their own conclusions about the protein's likely function. To date, three predictions submitted to COMBREX by computational teams have been successfully validated by experimental assays (manuscript in preparation).

## Supporting Information

Figure S1 Pie charts showing relative sequence similarity of uncharacterized proteins in COMBREX to experimentally characterized (*green*) proteins. (A) *Blue* proteins. (B) *Black* proteins. Within each pie, proteins are divided into those that exhibit “strong” similarity, “weak” similarity, or “no” similarity to characterized proteins. Strong similarity requires a BLASTP match of E≤1e-05 along with 80% sequence identity along 80% of the length of both query and hit, and identical composition of domains as determined by Pfam; these criteria are used by COMBREX to generate predictions, so all such genes are *blue* by definition. Weak similarity requires only a BLASTP match of E≤1e-05, with the aligned region covering 80% of the length of both query and hit, with no other constraints; weak similarity is not directly used to generate predictions by COMBREX, hence a small portion of *black* proteins satisfy these criteria. Conversely, as predictions for *blue* proteins come from a number of sources, a significant number of *blue* proteins do not satisfy either the strong or weak sequence similarity criteria and are categorized as having no similarity to any characterized protein.
(TIF)

Figure S2 Number of clusters as a function of cluster size. Clusters are broken down into three types based on the functional status of their component proteins: clusters containing ≥1 experimentally characterized (*green*) gene are represented by the green line; clusters containing no experimentally characterized proteins but ≥1 protein with a predicted function (*blue*) are represented by the blue line; clusters where no proteins have either a characterized or predicted function are represented by the black line. Cluster sizes are grouped with a bin size of 10, and in several instances a pseudocount of 1 was added to 0 values to ensure continuous lines in logarithmic scale.
(TIF)

Figure S3 Domain composition of proteins in COMBREX. All COMBREX proteins were clustered into groups based on identical domain composition. Along the *x*-axis, groups are separated based on the number of annotated Pfam domains per protein (as defined by Pfam). (A) Histogram, where the green portion of each bar indicates the number of proteins that have identical domain composition to an experimentally characterized (*green*) protein, the blue portion those that have identical domain composition to a protein with a predicted function (*blue*), and the black portion all others. (B) Same data shown in logarithmic scale, where the green, blue, and black lines represent the sizes of the green, blue, and black portions of the histogram bars in part A.
(TIF)

Figure S4 Flowchart of GSDB construction. Source information includes external databases such as UniProtKB and other databases (“Source DBs”), and genes nominated by users via the COMBREX website. All entries originating outside of UniProtKB must be assigned a unique UniProtKB accession number before entry into the process. All candidates with a UniProtKB accession number enter the GSDB curation queue. After examination by COMBREX curators, genes may be accepted into the GSDB if they meet the Gold Standard criteria. Those not accepted are returned to UniProt for additional research, and so that the UniProtKB records may be appropriately updated if necessary. Contents of the GSDB are visible in COMBREX as *green* proteins, where curated Gold Standard proteins are labeled with a gold “G,” and proteins awaiting curation are not. Proteins failing the curation process join the *blue* set, like all other proteins with no definitive experimental information.
(TIF)

Table S1 Summary of proteins examined by COMBREX-funded projects.
(XLSX)

Table S2 Association of structural data with uncharacterized proteins.
(DOC)

Table S3 Format of functional descriptions in COMBREX.
(DOC)

Table S4 Free-text strings analyzed by GOCat.
(DOC)

Table S5 Function predictions submitted to COMBREX by external groups.
(DOC)

Text S1 More detailed description of the following topics: selected COMBREX-funded experimental results; functional inference from existing experimental information; use of structured vocabulary; and prioritization of genes for experimental characterization. Materials and Methods, including the following topics: the COMBREX website; functional status of genes; clustering of genes; semantic analysis of free-text functional descriptions; and calculation of sequence distances within clusters.
(DOC)

## Abbreviations

COMBREX: COMputational BRidges to EXperiments
EC: Enzyme Commission
GO: Gene Ontology
GSDB: Gold Standard Database
