# Supplementary figures and images for: The COMBREX Project: Design, Methodology, and Initial Results
Source: PLoS Biol. 2013 Aug 27;11(8):e1001638. doi: 10.1371/journal.pbio.1001638 (PMC3754883; doi:10.1371/journal.pbio.1001638)

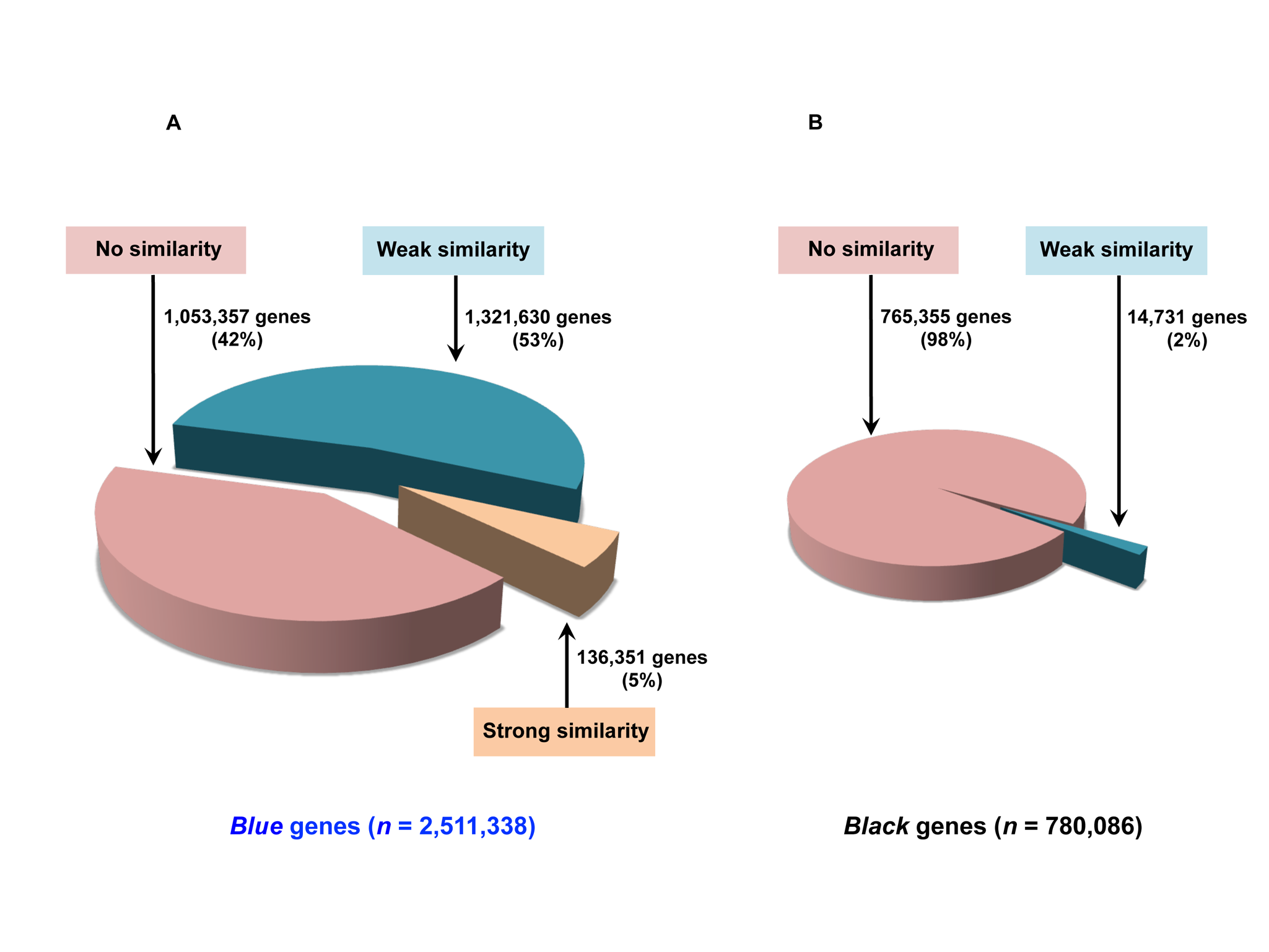

Supplement: Figure S1 — Pie charts showing relative sequence similarity of uncharacterized proteins in COMBREX to experimentally characterized (green) proteins. (A) Blue proteins. (B) Black proteins. Within each pie, proteins are divided into those that exhibit “strong” similarity, “weak” similarity, or “no” similarity to characterized proteins. Strong similarity requires a BLASTP match of E≤1e-05 along with 80% sequence identity along 80% of the length of both query and hit, and identical composition of domains as determined by Pfam; these criteria are used by COMBREX to generate predictions, so all such genes are blue by definition. Weak similarity requires only a BLASTP match of E≤1e-05, with the aligned region covering 80% of the length of both query and hit, with no other constraints; weak similarity is not directly used to generate predictions by COMBREX, hence a small portion of black proteins satisfy these criteria. Conversely, as predictions for blue proteins come from a number of sources, a significant number of blue proteins do not satisfy either the strong or weak sequence similarity criteria and are categorized as having no similarity to any characterized protein. (TIF) [file pbio.1001638.s001.tif]

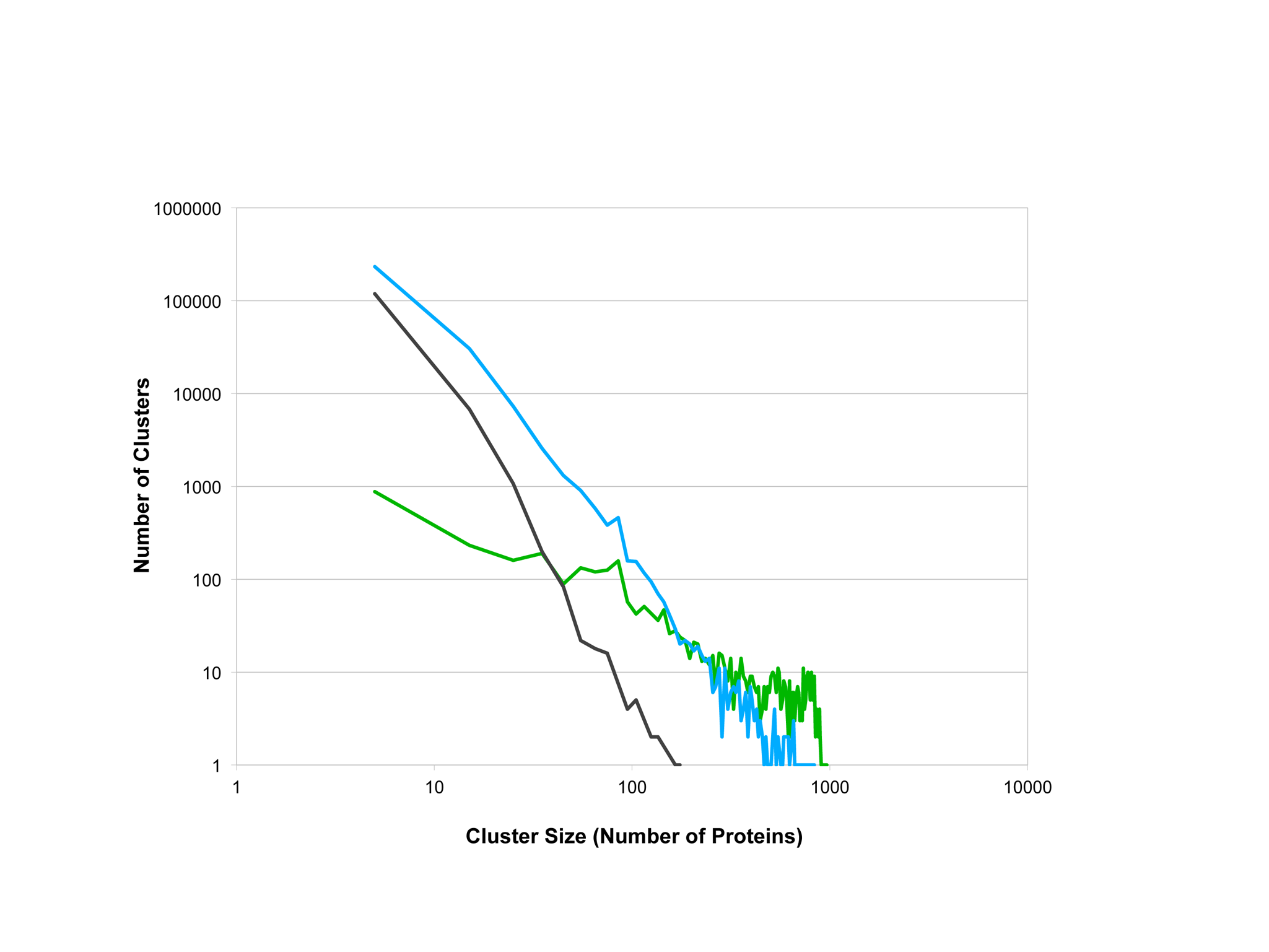

Supplement: Figure S2 — Number of clusters as a function of cluster size. Clusters are broken down into three types based on the functional status of their component proteins: clusters containing ≥1 experimentally characterized (green) gene are represented by the green line; clusters containing no experimentally characterized proteins but ≥1 protein with a predicted function (blue) are represented by the blue line; clusters where no proteins have either a characterized or predicted function are represented by the black line. Cluster sizes are grouped with a bin size of 10, and in several instances a pseudocount of 1 was added to 0 values to ensure continuous lines in logarithmic scale. (TIF) [file pbio.1001638.s002.tif]

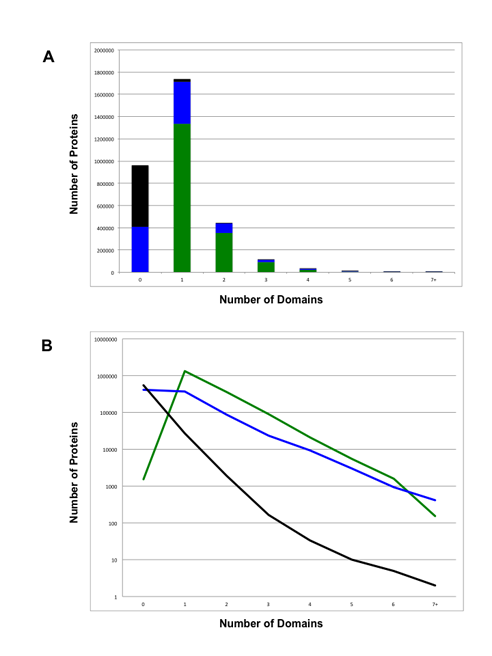

Supplement: Figure S3 — Domain composition of proteins in COMBREX. All COMBREX proteins were clustered into groups based on identical domain composition. Along the x-axis, groups are separated based on the number of annotated Pfam domains per protein (as defined by Pfam). (A) Histogram, where the green portion of each bar indicates the number of proteins that have identical domain composition to an experimentally characterized (green) protein, the blue portion those that have identical domain composition to a protein with a predicted function (blue), and the black portion all others. (B) Same data shown in logarithmic scale, where the green, blue, and black lines represent the sizes of the green, blue, and black portions of the histogram bars in part A. (TIF) [file pbio.1001638.s003.tif]

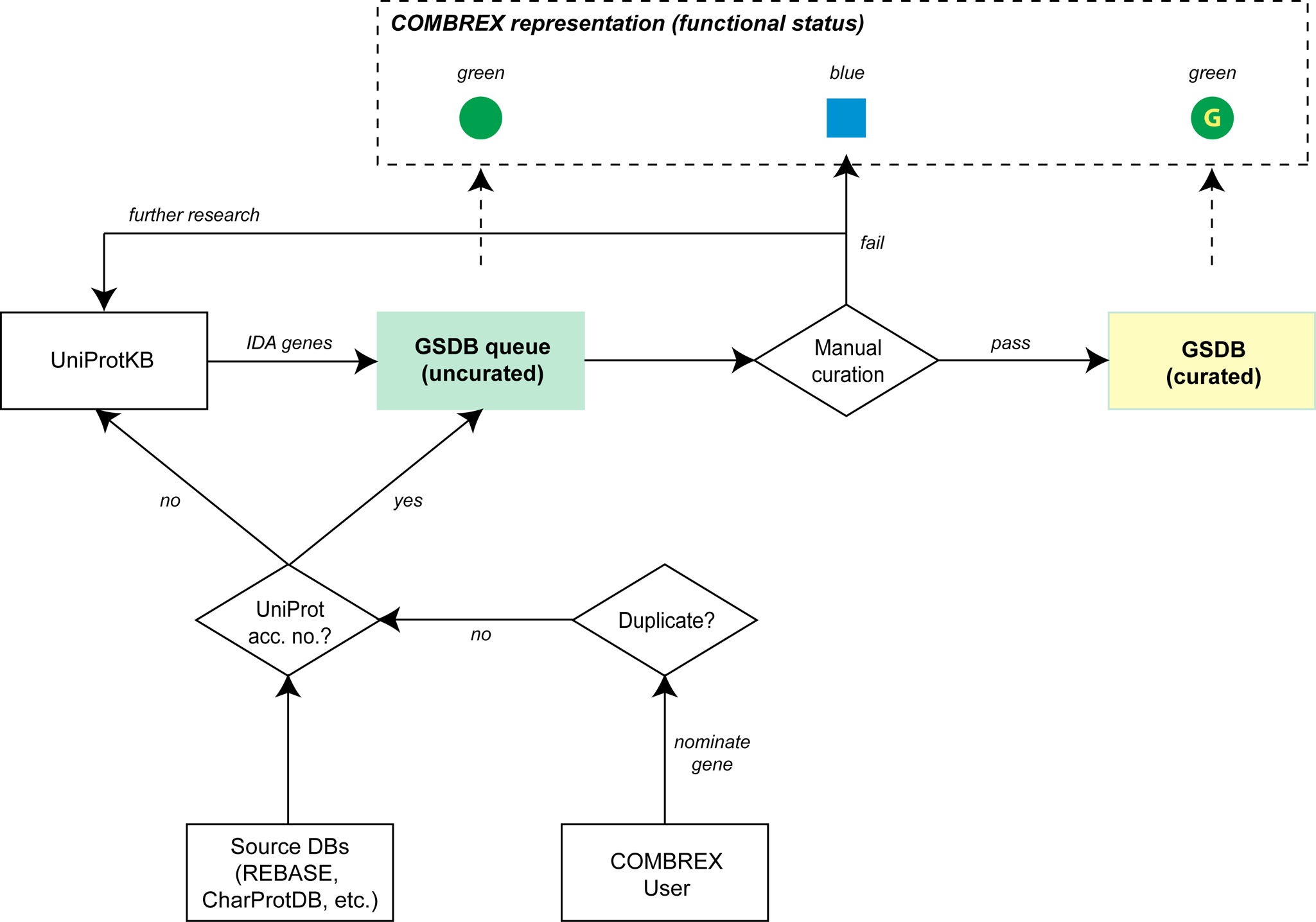

Supplement: Figure S4 — Flowchart of GSDB construction. Source information includes external databases such as UniProtKB and other databases (“Source DBs”), and genes nominated by users via the COMBREX website. All entries originating outside of UniProtKB must be assigned a unique UniProtKB accession number before entry into the process. All candidates with a UniProtKB accession number enter the GSDB curation queue. After examination by COMBREX curators, genes may be accepted into the GSDB if they meet the Gold Standard criteria. Those not accepted are returned to UniProt for additional research, and so that the UniProtKB records may be appropriately updated if necessary. Contents of the GSDB are visible in COMBREX as green proteins, where curated Gold Standard proteins are labeled with a gold “G,” and proteins awaiting curation are not. Proteins failing the curation process join the blue set, like all other proteins with no definitive experimental information. (TIF) [file pbio.1001638.s004.tif]
