# Supplementary material for: The COMBREX Project: Design, Methodology, and Initial Results
Source: PLoS Biol. 2013 Aug 27;11(8):e1001638. doi: 10.1371/journal.pbio.1001638 (PMC3754883; doi:10.1371/journal.pbio.1001638)
Supplement: Table S2 — Association of structural data with uncharacterized proteins. (DOC) [file pbio.1001638.s006.doc]

Table S2. Association of structural data with uncharacterized proteins.

| Functional status | No. Proteins | No. Structures | No. Structural Associations^a^ | | Associations per Structure^b^ | | % Protein Space Covered by (Structures) or Associations^c^ | |
| --- | --- | --- | --- | --- | --- | --- | --- | --- |
|  |  |  | Cluster | Super-cluster | Cluster | Super-cluster | Cluster | Super-cluster |
| *green* | 10,969 | 3,277 | 369,204 | 478,556 | 113 | 146 | (29.9) | (29.9) |
| *blue* | 2,511,338 | 8,277 | 102,405 | 208,451 | 12.4 | 25.2 | 18.3 | 26.9 |
| *black* | 780,086 | 1,102 | 6,739 | 13,107 | 6.12 | 11.9 | 0.864 | 1.68 |

^a^ Number of structural associations are the number of genes in the same cluster or super-cluster as ≥1 structurally characterized gene of the indicated functional status.

^b^ Number of associations per structure = (number of structural associations) / (number of structures).

^c^ Percentage of gene space calculated as follows. For *green*, number = (total number of structures) / (number of proteins); shown in parentheses because it is independent of cluster or super-cluster data. For *blue*, number = (number of structural associations for *green+blue* – number of structures for *green+blue*) / (total number of proteins). For *black*, number = (number of structural associations) / (total number of proteins).
