# Supplementary material for: The COMBREX Project: Design, Methodology, and Initial Results
Source: PLoS Biol. 2013 Aug 27;11(8):e1001638. doi: 10.1371/journal.pbio.1001638 (PMC3754883; doi:10.1371/journal.pbio.1001638)
Supplement: Table S3 — Format of functional descriptions in COMBREX. (DOC) [file pbio.1001638.s007.doc]

Table S3. Format of functional descriptions in COMBREX.

| **Source** | **Number of Predictions** ^a^ | | | |
| --- | --- | --- | --- | --- |
|  | **Free Text**^b^ | **GO (MF)**^c^ | **EC** | ***Total*** |
| RefSeq | 2,169,419 | 0 | 407,070 | 2,576,489 |
| ProtClustDB | 2,138,532 | 0 | 279,592 | 2,418,124 |
| BRENDA | 0 | 0 | 399,394 | 399,394 |
| GO | 0 | 5,091,938 | 0 | 5,091,938 |
| Contributors^d^ | 72 | 3,194 | 7,781 | 11,047 |
| *Total* | 4,308,023 | 5,095,132 | 1,093,837 | 10,496,992 |

^a^ Predictions are considered <*gene*, *description*> pairs, so the same description may be counted multiple times if it is applied to multiple genes. Furthermore, one gene often has multiple predictions associated with it, from various sources.

^b^ Free text predictions exclude only those considered uninformative based on semantic analysis (Materials and Methods).

^c^ GO predictions include Molecular Function predictions only, and exclude the following highly general terms: GO:0003674 (molecular function), GO:0003824 (catalytic activity), GO:0005488 (binding).

^d^ Contributors' predictions include all those submitted directly by COMBREX users and collaborators.
