# Supplementary material for: The COMBREX Project: Design, Methodology, and Initial Results
Source: PLoS Biol. 2013 Aug 27;11(8):e1001638. doi: 10.1371/journal.pbio.1001638 (PMC3754883; doi:10.1371/journal.pbio.1001638)
Supplement: Table S4 — Free-text strings analyzed by GOCat. (DOC) [file pbio.1001638.s008.doc]

Table S4. Free-text strings analyzed by GOCat.

|  | **Total Clusters** | **Total Non-Redundant Descriptions** |
| --- | --- | --- |
| Starting set | 409,016 | 61,182 |
| Exact match to GO | 30,061 | 1913 |
| Uninformative | 151,633 | 8828 |
| Exact match AND uninformative | 81 | 4 |
| Not exact match AND informative | 227,403 | 53,928 |
