# Supplementary material for: The COMBREX Project: Design, Methodology, and Initial Results
Source: PLoS Biol. 2013 Aug 27;11(8):e1001638. doi: 10.1371/journal.pbio.1001638 (PMC3754883; doi:10.1371/journal.pbio.1001638)
Supplement: Table S5 — Function predictions submitted to COMBREX by external groups. (DOC) [file pbio.1001638.s009.doc]

Table S5. Function predictions submitted to COMBREX by external groups.

| **Name** | **Methodology** | **No. Genes** | **Format** | **Reference(s)** |
| --- | --- | --- | --- | --- |
| VisANT-GF | Identification of functionally linked genes using gene fusion (Rosetta Stone method) | 907 | Gene pairs |  |
| VisANT-PP | Identification of functionally linked genes using phylogenetic profiling | 1538 | Gene pairs |  |
| VisANT-CN | Identification of functionally linked genes using conserved gene neighborhood | 1833 | Gene pairs |  |
| OperonDB | Identification of functionally linked genes based on predicted operon structure | 1332 | Gene pairs |  |
| DME | Data Mining of Enzymes (prediction of catalytic function based on conserved active site peptide motifs) | 76 | EC |  |
| CGC | Context-based Genomic Correlations (Gibbs sampling of metabolic networks using sequence-based and context-based correlations) | 7065 | EC |  |
| PHOG | PhyloFacts Orthology Group (prediction of super-orthologs using evolutionary analysis of protein family phylogenies) | 774 | EC, GO |  |
| MTDB | Methyltransferase database (hand curation of methyltransferase orthologous families) | 44 | Text |  |
| F_420_/FMN | Prediction of F_420_- and FMN-dependent proteins in *Mycobacterium smegmatis* using HMMs and phylogenetic profiling | 28 | Text |  |

**References**

1. Marcotte EM, Pellegrini M, Ng HL, Rice DW, Yeates TO, et al. (1999) Detecting protein function and protein-protein interactions from genome sequences. Science 285: 751-753.

2. Yanai I, Derti A, DeLisi C (2001) Genes linked by fusion events are generally of the same functional category: a systematic analysis of 30 microbial genomes. Proceedings of the National Academy of Sciences of the United States of America 98: 7940-7945.

3. Hu Z, Hung JH, Wang Y, Chang YC, Huang CL, et al. (2009) VisANT 3.5: multi-scale network visualization, analysis and inference based on the gene ontology. Nucleic Acids Res 37: W115-121.

4. Pellegrini M, Marcotte EM, Thompson MJ, Eisenberg D, Yeates TO (1999) Assigning protein functions by comparative genome analysis: protein phylogenetic profiles. Proc Natl Acad Sci U S A 96: 4285-4288.

5. Wu J, Kasif S, DeLisi C (2003) Identification of functional links between genes using phylogenetic profiles. Bioinformatics 19: 1524-1530.

6. Dandekar T, Snel B, Huynen M, Bork P (1998) Conservation of gene order: a fingerprint of proteins that physically interact. Trends Biochem Sci 23: 324-328.

7. Overbeek R, Fonstein M, D'Souza M, Pusch GD, Maltsev N (1999) The use of gene clusters to infer functional coupling. Proc Natl Acad Sci U S A 96: 2896-2901.

8. Pertea M, Ayanbule K, Smedinghoff M, Salzberg SL (2009) OperonDB: a comprehensive database of predicted operons in microbial genomes. Nucleic Acids Res 37: D479-482.

9. Weingart U, Lavi Y, Horn D (2009) Data mining of enzymes using specific peptides. BMC Bioinformatics 10: 446.

10. Kunik V, Meroz Y, Solan Z, Sandbank B, Weingart U, et al. (2007) Functional representation of enzymes by specific peptides. PLoS Comput Biol 3: e167.

11. Hsiao TL, Revelles O, Chen L, Sauer U, Vitkup D Automatic policing of biochemical annotations using genomic correlations. Nat Chem Biol 6: 34-40.

12. Chen L, Vitkup D (2006) Predicting genes for orphan metabolic activities using phylogenetic profiles. Genome Biol 7: R17.

13. Datta RS, Meacham C, Samad B, Neyer C, Sjolander K (2009) Berkeley PHOG: PhyloFacts orthology group prediction web server. Nucleic Acids Res 37: W84-89.

14. Anton BP (2010) unpublished.

15. Selengut JD, Haft DH Unexpected abundance of coenzyme F(420)-dependent enzymes in Mycobacterium tuberculosis and other actinobacteria. J Bacteriol 192: 5788-5798.
