# Supplementary material for: The COMBREX Project: Design, Methodology, and Initial Results
Source: PLoS Biol. 2013 Aug 27;11(8):e1001638. doi: 10.1371/journal.pbio.1001638 (PMC3754883; doi:10.1371/journal.pbio.1001638)
Supplement: Text S1 — More detailed description of the following topics: selected COMBREX-funded experimental results; functional inference from existing experimental information; use of structured vocabulary; and prioritization of genes for experimental characterization. Materials and Methods, including the following topics: the COMBREX website; functional status of genes; clustering of genes; semantic analysis of free-text functional descriptions; and calculation of sequence distances within clusters. (DOC) [file pbio.1001638.s010.doc]

**The COMBREX Project: Design, Methodology, and Initial Results**

(Running Head: “COMBREX: Design, Methodology, and Initial Results”)

**SUPPLEMENTARY INFORMATION**

COMBREX-FUNDED EXPERIMENTAL RESULTS

Table S1 provides a complete list of the 140 proteins tested in COMBREX-funded and COMBREX-associated experimental efforts. Below, we highlight the results of three COMBREX grants as examples.

A novel epimerase from *Thermotoga maritima* MSB8. The gene TM0440 from *T. maritima*MSB8 belongs to a widely distributed family of proteins (the cluster contains 16 members, including both bacterial and archaeal proteins), none of which had any associated functional predictions previously. This family had no recognizable protein domains and no significant sequence similarity to any experimentally characterized protein. The investigating team (at Sanford-Burnham Medical Research Institute, led by A. Osterman) themselves predicted that TM0440 acted as a novel epimerase interconverting tagaturonate and fructuronate based on genome context analysis (operon and regulon structure, and metabolic gap filling, as described elsewhere ). This proposed enzymatic activity was unprecedented, and it does not currently have an assigned EC number. The investigators cloned and expressed TM0440 (now called UxaE) and confirmed the predicted activity *in vitro*, with detection by gas chromatography–mass spectrometry. The TM0441 gene, located in the same operon as TM0440, encodes a previously uncharacterized enzyme distantly homologous to 5-keto-D-gluconate reductase. TM0441 (now called UxaD) was also cloned and expressed and found to catalyze the NADPH-dependent reduction of D-fructuronate to D-mannonate. As a result, the specific functions of both UxaE and UxaD were assigned within the pectin utilization pathway of *T. maritima*. Details of this work have recently been described elsewhere .

Investigation of the 2-nitropropane dioxygenase family. Sixty years ago extracts from *Neurospora crassa* were reported to oxidize nitroethane with the release of aldehyde and nitrite . An enzyme with similar properties was purified from *Cyberlindnera mrakii* (formerly *Hansenula mrakii*) and given the name 2-nitropropane dioxgenase based on activities measured under non-optimal conditions. The name was extended to the enzyme from *N. crassa* even though the physiological role of the enzymes were unknown and biochemical characterization of the purified enzyme did not occur for another 30 years . The sequence of the gene encoding the enzyme from *C. mrakii* was the first such sequence deposited in EMBL , and it became the basis for several thousand genes from a total of 147 protein clusters to be annotated as 2-nitropropane dioxygenases. Recent biochemical recharacterization of the enzymes from *C. mrakii* and *N. crassa* by the investigators associated with COMBREX revealed that the true physiological substrates of the enzymes are alkyl nitronates rather than nitroalkanes , and the EC number and official enzyme name has been changed to nitronate monooxygenase. The amino acid sequences of enzymes that enable bacteria to grow on 3-nitropropionic acid but not on nitroalkanes such as 2-nitropropane are also nitronate monooxygenases . The joint investigators (Jim Spain at Georgia Institute of Technology and Giovanni Gadda at Georgia State University) have further characterized the enzyme from *N. crassa* and found that the true physiological substrate is propionate 3-nitronate rather than an alkyl nitronate . They are examining 10 genes from 8 clusters and 5 bacterial species with the aim of establishing the activities of the enzymes toward a range of nitroaliphatic compounds including 3-nitropropionic acid, propionate-3-nitronate, and nitroethane. Results to date indicate that many if not all of the “2-nitropropane dioxygenases” are misannotated and that the error has been widely propagated. The findings illustrate the idea that detecting a function and establishing the physiological roles of enzymes are substantially different and the latter is much more biologically important. This work is ongoing, but a successful outcome will improve the predictions for the many genes in this large family.

Restriction endonuclease discoveries. Because they evolve rapidly, restriction endonucleases can often be difficult to predict accurately by sequence similarity. The investigators (A. Fomenkov, R. D. Morgan, and S.-Y. Xu at New England Biolabs) tested more than 40 predicted endonucleases and DNA methyltransferases, confirmed enzymatic activity in 35, and in most cases determined the recognition sequence for cleavage or methylation. These enzymes include Type IIG enzymes (endonuclease and methyltransferase activities reside on the same polypeptide) for which several novel recognition sites have been identified, several Type III enzymes that all show novel recognition sites, and 10 methyl-dependent enzymes with novel activities, including one specifically recognizing 5-hydroxymethylcytosine. In addition, the investigators are researching a family of genes related to HpaII (which recognizes unmethylated CCGG) that lack a companion methyltransferase gene, suggesting they may encode methyl-dependent enzymes as well. Results from this work are described in Table S1 and are available via REBASE ([http://rebase.neb.com](http://rebase.neb.com/)) . The characterization of these genes will improve predictions for many restriction enzyme genes, which are widespread in bacterial genomes.

FUNCTIONAL INFERENCE USING EXISTING EXPERIMENTAL INFORMATION

Sequence information. Figure S1A shows the fractions of *blue* proteins that are related through defined sequence-similarity measures to at least one experimentally characterized protein (see Materials and Methods), while Figure S1B does the same for *black* proteins. "Strong" sequence similarity is used as the basis for functional prediction by COMBREX, in which the uncharacterized protein is predicted to have the biochemical function of the characterized protein. Proteins with such predictions form 5% of the overall *blue* set (Figure S1A). "Weak" sequence similarity, while potentially useful for making functional inferences, is not a criterion for generating explicit predictions in COMBREX. Although the fraction of experimentally characterized genes is relatively small (Figure 2 in the Main Text), more than half of all *blue* genes are related through at least weak sequence similarity to these few characterized genes (Figure S1A). This can be partially explained by the fact that genes that have been experimentally characterized tend to be highly conserved and/or from large gene families (Figure S2, green line). Genes with no meaningful predictions, by contrast, tend to come from smaller families (Figure S2, black line).

Domain information. When the function of a protein as a whole is unknown, or at least annotated as such, the function of one or more of its domains in other contexts may still be deduced. Of the protein sequences in COMBREX, 2,337,489 (71%) contain one or more domains as defined by Pfam v24.0 . Since proteins sharing the same set of domains are statistically likely to share similar overall functions , we determined which of these proteins shared the same set of domains (regardless of order) with an experimentally characterized (*green*) protein, or failing that, with one or more proteins with a predicted function (*blue*). Figure S3A plots these figures as a function of the number of domains in the protein. As may be expected, particularly for microbial proteins, the number of proteins decreases exponentially as the number of domains increases (Figure S3B). However, the fraction of all genes represented in Figure S3 that share identical domain composition with an experimentally characterized protein (green portions of each bar) is a surprising 55%. This fraction is largely independent of the number of domains per protein.

Association of Proteins With Structural Data. Structures of more than 8,500 bacterial proteins have been solved and deposited in PDB (as represented in UniProtKB, accessed January 2010). Because proteins belonging to the same cluster, and to a slightly lesser extent the same super-cluster, are highly sequence-similar, they are expected to have highly similar structures. If a cluster contains one or more members whose structures have been solved, that structural information can be associated with all other members of the cluster. (We are essentially "predicting" structure based on “cluster approximation,” without performing formal threading or homology modeling.) Table S2 shows the results of such an associative analysis, in which proteins are separated based on functional status. The 3,277 structures of *green* proteins can be associated, through shared cluster membership, with 369,204 proteins (including self-associations), or about 113 associations per structure. For *blue* and *black* proteins, the number of associations per structure drops considerably, to 12 and 6 respectively. Surprisingly, 18% of all *blue* proteins, but only 0.9% of all *black* proteins, can be associated in this way with a solved structure through shared cluster membership (Table S2). The poor coverage of the *black* protein space may again have to do with the relatively small size of clusters containing *black* proteins (Figure S2) or difficulty characterizing specific types of proteins (such as membrane proteins) both structurally and functionally. Finally, 30% of all *green* proteins have directly solved structures, which is not surprising since structural study is often focused on proteins of known function, and since structural and functional characterizations are often performed as part of the same study.

STRUCTURED VOCABULARY

In order to compare functional predictions in a large-scale, high throughput manner, one requires a computable, or machine-readable, format, namely structured vocabulary. While semantic analysis of free text descriptions is possible, in practice it is difficult due to the large number of possible synonymous terms and the general complexity of biochemical description. It is a goal of COMBREX to associate structured vocabulary terms with protein function predictions and annotation whenever possible, preferably through automated means. We are therefore striving to associate structured vocabulary terms with as many predictions in COMBREX as possible. Many predictions imported from public databases are exclusively in free text format. Table S3 shows the fractions of predictions in COMBREX with functional descriptions currently in the form of free-text descriptions, GO terms and EC numbers.

The vast majority of proteins in COMBREX belong to one of 409,016 clusters as assigned by ProtClustDB (Materials and Methods), each of which is associated with one of 64,664 non-redundant free-text functional descriptions (Table S4). A comparison of these descriptions with definitions of GO terms identified 1,913 exact string matches, so these clusters were automatically assigned the matching GO term. A further 8,823 text descriptions were identified as functionally uninformative (Materials and Methods) and excluded from further analysis. The remaining 53,928 terms (Table S4) were associated with likely GO terms using the predictive-model algorithm in the Gene Ontology Categorizer (GOCat; <http://eagl.unige.ch/GOCat/>) . GO terms scoring above 0.50 were retained as probable GO terms. This threshold retained, on average, five GO terms per input string, and this threshold was established because it provided a set of terms that best matched the GO terms assigned by UniProt for a test set consisting of genes from *H. pylori* 26695 genes (data not shown). We plan to perform similar GO-term associations using predictions from other sources such as RefSeq and UniProt, where such terms are not already available.

PRIORITIZATION OF GENES

Active learning. Major advances in the art and science of protein function prediction have been made in recent years, with methods based on pairwise sequence similarity , sequence profiles , protein families , evolutionary modeling , functional linkage , genomic context , gap-filling in metabolic networks , genomic role-filling , structural remote homology modeling and threading , protein-ligand docking , and combinations of these . All predictive methods, regardless of strategy, rely on existing experimental data to make inferences about uncharacterized proteins. The incorporation of new experimental data for one or more member proteins into the model allows for new or more accurate predictions for other members, thereby increasing the total information of the set. Predictive models learn the most about a set of proteins by the experiment that produces the maximum gain of information, and so identifying such experiments is of critical importance. A conceptual framework for this process is the subfield of prediction theory called *active learning* . In active learning, a recommendation function selects new data whose labeling (revealed, i.e., through experimental analysis) will maximally improve the capability of the prediction system. This is in contrast to standard prediction systems, which learn prediction rules and make predictions based on available data.

Active learning is an iterative process that attempts to optimize the choice of experiments at each iteration to maximize information gain. Our expectation is that experiments on a small number of carefully chosen proteins will provide maximal value once predictions from these results are extended to all other proteins. A simple, heuristic way of describing our application of active learning is, “choose a small set of proteins whose ‘distance’ to all other proteins in the set is minimized.” We plan to further formalize our approach using active learning frameworks governed by machine learning classifiers and have begun proof-of-concept experiments towards this end.

Prioritization criteria. The first criterion is the functional status of proteins assigned by COMBREX, with the rationale being to focus on those that have testable predicted functions but no associated experimental evidence [*blue* genes (Figure 2)]. Formally, this is because further confirmation of already verified functions adds little or no new information to the system, and because performing specific assays on proteins with no predicted function has a lower *a priori* chance of success. We expect that predictions within the *blue* category span a wide range of probabilities, from proteins that are 99% identical to experimentally characterized examples to those with low-probability evidence based on weak functional linkage (for example, members of operons), and a wide range of substrate specificities, from precise (e.g., *S-succinylglutathione hydrolase*) to general (e.g., *hydrolase*). We seek intuitively to focus efforts on predictions that are testable but not trivial.

Second, when recommending proteins to examine from within a large family (cluster), COMBREX recommends candidates based on two properties: genome of origin and position within the cluster. We have chosen two "focus organisms," *Escherichia coli* K-12 MG1655 and *Helicobacter pylori* 26695, for which we would like to obtain a large amount of experimental evidence, and we encourage confirming predictions from genes in these two strains. The former is an archetypal *E. coli* strain in which a large number of experimental characterizations have already been carried out, and so the goal is to continue building a "complete" understanding of the biochemical potential of a single genome. The latter is a type strain of *H. pylori*, an organism that is important to human health and disease, is evolutionarily distant from *E. coli*, and by contrast to *E. coli* has had comparatively few genes characterized biochemically. For the many clusters that contain members from neither of these genomes, the recommended gene is that with the shortest average sequence distance to all other members of the cluster, in an attempt to select a gene most likely to be representative of the family (see Methods, in Supplementary Information). Ideally, we encourage selection of several genes within a given family in order to confirm a common substrate or function and help define the boundaries where substrate specificity or function might change. One way to select such candidates would be by careful examination of the phylogenetic tree of cluster members (phylogenomic method) .

Third, we recommend proteins from larger protein families over those from smaller families under the assumption that experimental evidence is likely to indicate that such families are isofunctional. Such proteins are encoded by highly ‘conserved hypothetical’ genes, the subject of one published list of proposed experimental targets . We define a protein family as those belonging to the same super-cluster as defined by ProtClustDB (Materials and Methods).

Finally, we recognize that there are significant contributory factors to "importance" that are independent of family size or sequence similarity. Examples of such factors might include being a key member of a particular biochemical pathway, having a biochemical function not previously identified experimentally, indicating functional diversification within a family previously thought to be isofunctional, or being associated with a phenotype of interest. COMBREX does not want to exclude such factors from its funding decisions, and so it has sought community participation to identify potentially "important" proteins based on these kinds of subjective measures. Users are encouraged to designate specific proteins with predicted functions as being of high priority to experimentally characterize.

MATERIALS AND METHODS

The COMBREX website. COMBREX can be freely accessed at [http://combrex.bu.edu](http://combrex.bu.edu/). Some participatory actions such as applying for funding or submitting information to COMBREX require registration. The GSDB (with the exception of its eukaryotic entries) is fully contained within COMBREX, and genes in the GSDB can be selectively accessed using COMBREX's advanced search features. Genes in the COMBREX Database can be accessed through NCBI’s Entrez Gene database via their LinkOut service in addition to through the COMBREX website directly. The analyses described in this work were performed on or before June 29, 2012, with COMBREX including 3,299,274 genes grouped in 409,016 clusters and data from 1,033 completely sequenced genomes.

Functional status of genes. The COMBREX Database contains over 3 million individual genes. In order to quickly and intuitively convey the evidence for any gene's function, we have adopted a color and symbol coding system that is modeled after those used on North American ski slopes to indicate the expected level of difficulty of experimental testing (Figure 2): a gene whose function is already known by experimental evidence should be "easy" to confirm (since a model for the correct experiment has already been established), a gene whose function has been predicted but not yet demonstrated might be of "moderate" difficulty, while experimental exploration of a gene with no predicted function is self-evidently "difficult". Among the *green* set, those proteins that have met the curation standards for the GSDB are indicated (Figure 2). The number of genes in each category is also indicated in Figure 2. We will use italics throughout this work to indicate where a color is used to indicate functional status.

Clustering of genes. Genes represented in COMBREX come largely from complete genome sequences of bacteria and archaea. These genomes include many groups of closely related strains or species, so there are many groups of genes of highly similar or even identical sequence present in the database. As an initial organizational principle and to reduce this redundancy, COMBREX has adopted the clustering system developed by the NCBI Protein Clusters Database (ProtClustDB) , which groups mutually highly related protein sequences using an algorithm which includes BLAST analysis. Some ProtClustDB clusters are curated, and the associated cluster descriptions are considered functional predictions for their member genes by COMBREX; however, the majority of clusters are uncurated. ProtClustDB also defines the concept of "related clusters", which are pairs of highly similar clusters . COMBREX allows for grouping of cliques of mutually related clusters into "superclusters." These groupings may be useful in cases where clusters are over-split with respect to a particular protein family. Unless otherwise stated, use of the term "cluster" in this work refers specifically to a particular gene group as defined by ProtClustDB. Many other methods of grouping microbial proteins into orthologous or homologous families have been devised, including TIGRFAMs , FIGfams , PANTHER , eggNOG , PhyloFacts , PHOG , OMA , OrthoMCL-DB , and others. In the future we plan to employ such alternative protein family groupings so that users can employ their favored grouping or toggle between groupings to compare results. We also anticipate grouping proteins based on neighborhoods in functional linkage networks .

COMBREX also includes genes from various sources that were not subject to clustering by ProtClustDB. Many GSDB genes fall into this category, since many experiments have been performed on proteins from organisms whose genomes have not been completely sequenced. Although many of these genes are highly similar to members of a particular cluster, COMBREX has avoided explicitly assigning these proteins to clusters for consistency with ProtClustDB.

Semantic analysis of free-text functional descriptions. In order to distinguish free-text annotations that contain useful functional predictions from those that are not suggestive of any specific function, we use a simple, automated semantic analysis protocol (ALM and BPA, unpublished). The text string is split into component tokens, each token is compared to a set of terms that do not contribute to functional description (examples: *hypothetical*, *element*, *protein*, *small*, *family*, four-letter gene symbols, etc.), and then each matching token is removed from the string. Our current "dictionary" of such terms, developed empirically from manual examination of representative descriptions, contains over 80 terms. If the string is empty after this step, the annotation is considered uninformative; otherwise, it is considered a functional prediction.

Sequence distances within clusters. The tree-based "sequence distance" that we employ measures the degree of similarity between two protein sequences, with shorter distances indicating greater similarities. For a given cluster, we simultaneously calculate the distances between all possible pairs of member sequences as follows. We first perform a multiple sequence alignment of all sequences in the cluster using MUSCLE . We then calculate a distance matrix for the alignment using the protdist program in PHYLIP , under the Jones-Taylor-Thornton model of amino acid substitution . The matrix entry for any pair of sequences is the "sequence distance." For each protein in a cluster, we calculate the average distance to all other proteins as well as the distance(s) to any experimentally validated protein(s) in the cluster. We plot the distributions of these distances.

**REFERENCES**

1. Yang C, Rodionov DA, Li X, Laikova ON, Gelfand MS, et al. (2006) Comparative genomics and experimental characterization of N-acetylglucosamine utilization pathway of Shewanella oneidensis. J Biol Chem 281: 29872-29885.

2. Yang C, Rodionov DA, Rodionova IA, Li X, Osterman AL (2008) Glycerate 2-kinase of Thermotoga maritima and genomic reconstruction of related metabolic pathways. J Bacteriol 190: 1773-1782.

3. Rodionov DA, Yang C, Li X, Rodionova IA, Wang Y, et al. Genomic encyclopedia of sugar utilization pathways in the Shewanella genus. BMC Genomics 11: 494.

4. Rodionova IA, Scott DA, Grishin NV, Osterman AL, Rodionov DA (2012) Tagaturonate-fructuronate epimerase UxaE, a novel enzyme in the hexuronate catabolic network in Thermotoga maritima. Environ Microbiol: accepted.

5. Little HN (1951) Oxidation of nitroethane by extracts from Neurospora. The Journal of biological chemistry 193: 347-358.

6. Francis K, Russell B, Gadda G (2005) Involvement of a flavosemiquinone in the enzymatic oxidation of nitroalkanes catalyzed by 2-nitropropane dioxygenase. The Journal of biological chemistry 280: 5195-5204.

7. Tchorzewski M, Kurihara T, Esaki N, Soda K (1994) Unique primary structure of 2-nitropropane dioxygenase from Hansenula mrakii. European journal of biochemistry / FEBS 226: 841-846.

8. Gadda G, Francis K (2010) Nitronate monooxygenase, a model for anionic flavin semiquinone intermediates in oxidative catalysis. Archives of biochemistry and biophysics 493: 53-61.

9. Nishino SF, Shin KA, Payne RB, Spain JC (2010) Growth of bacteria on 3-nitropropionic acid as a sole source of carbon, nitrogen, and energy. Applied and environmental microbiology 76: 3590-3598.

10. Francis K, Nishino SF, Spain JC, Gadda G (2012) A novel activity for fungal nitronate monooxygenase: detoxification of the metabolic inhibitor propionate-3-nitronate. Archives of biochemistry and biophysics 521: 84-89.

11. Roberts RJ, Vincze T, Posfai J, Macelis D REBASE--a database for DNA restriction and modification: enzymes, genes and genomes. Nucleic Acids Res 38: D234-236.

12. Finn RD, Mistry J, Tate J, Coggill P, Heger A, et al. The Pfam protein families database. Nucleic Acids Res 38: D211-222.

13. Hegyi H, Gerstein M (2001) Annotation transfer for genomics: measuring functional divergence in multi-domain proteins. Genome research 11: 1632-1640.

14. Ruch P (2006) Automatic assignment of biomedical categories: toward a generic approach. Bioinformatics 22: 658-664.

15. Altschul SF, Madden TL, Schaffer AA, Zhang J, Zhang Z, et al. (1997) Gapped BLAST and PSI-BLAST: a new generation of protein database search programs. Nucleic acids research 25: 3389-3402.

16. Sammut SJ, Finn RD, Bateman A (2008) Pfam 10 years on: 10,000 families and still growing. Briefings in bioinformatics 9: 210-219.

17. Hunter S, Apweiler R, Attwood TK, Bairoch A, Bateman A, et al. (2009) InterPro: the integrative protein signature database. Nucleic acids research 37: D211-215.

18. Datta RS, Meacham C, Samad B, Neyer C, Sjolander K (2009) Berkeley PHOG: PhyloFacts orthology group prediction web server. Nucleic Acids Res 37: W84-89.

19. Engelhardt BE, Jordan MI, Srouji JR, Brenner SE (2011) Genome-scale phylogenetic function annotation of large and diverse protein families. Genome research 21: 1969-1980.

20. Letovsky S, Kasif S (2003) Predicting protein function from protein/protein interaction data: a probabilistic approach. Bioinformatics 19 Suppl 1: i197-204.

21. Murali TM, Wu CJ, Kasif S (2006) The art of gene function prediction. Nature biotechnology 24: 1474-1475; author reply 1475-1476.

22. Galperin MY, Koonin EV (2000) Who's your neighbor? New computational approaches for functional genomics. Nature biotechnology 18: 609-613.

23. Marcotte EM, Pellegrini M, Ng HL, Rice DW, Yeates TO, et al. (1999) Detecting protein function and protein-protein interactions from genome sequences. Science 285: 751-753.

24. Thiele I, Palsson BO (2010) A protocol for generating a high-quality genome-scale metabolic reconstruction. Nature protocols 5: 93-121.

25. Keseler IM, Collado-Vides J, Santos-Zavaleta A, Peralta-Gil M, Gama-Castro S, et al. (2011) EcoCyc: a comprehensive database of Escherichia coli biology. Nucleic acids research 39: D583-590.

26. Roy A, Kucukural A, Zhang Y (2010) I-TASSER: a unified platform for automated protein structure and function prediction. Nature protocols 5: 725-738.

27. Morris GM, Huey R, Lindstrom W, Sanner MF, Belew RK, et al. (2009) AutoDock4 and AutoDockTools4: Automated docking with selective receptor flexibility. Journal of computational chemistry 30: 2785-2791.

28. Keiser MJ, Setola V, Irwin JJ, Laggner C, Abbas AI, et al. (2009) Predicting new molecular targets for known drugs. Nature 462: 175-181.

29. Marcotte EM, Pellegrini M, Thompson MJ, Yeates TO, Eisenberg D (1999) A combined algorithm for genome-wide prediction of protein function. Nature 402: 83-86.

30. King RD, Whelan KE, Jones FM, Reiser PG, Bryant CH, et al. (2004) Functional genomic hypothesis generation and experimentation by a robot scientist. Nature 427: 247-252.

31. Angluin D (1988) Queries and concept learning. Machine Learning 2: 319-342.

32. Cohn D, Atlas L, Ladner R (1994) Improving generalization with active learning. Machine Learning 15: 201-221.

33. Galperin MY, Koonin EV (2004) 'Conserved hypothetical' proteins: prioritization of targets for experimental study. Nucleic acids research 32: 5452-5463.

34. Klimke W, Agarwala R, Badretdin A, Chetvernin S, Ciufo S, et al. (2009) The National Center for Biotechnology Information's Protein Clusters Database. Nucleic Acids Res 37: D216-223.

35. Selengut JD, Haft DH, Davidsen T, Ganapathy A, Gwinn-Giglio M, et al. (2007) TIGRFAMs and Genome Properties: tools for the assignment of molecular function and biological process in prokaryotic genomes. Nucleic acids research 35: D260-264.

36. Meyer F, Overbeek R, Rodriguez A (2009) FIGfams: yet another set of protein families. Nucleic acids research 37: 6643-6654.

37. Mi H, Dong Q, Muruganujan A, Gaudet P, Lewis S, et al. (2010) PANTHER version 7: improved phylogenetic trees, orthologs and collaboration with the Gene Ontology Consortium. Nucleic acids research 38: D204-210.

38. Powell S, Szklarczyk D, Trachana K, Roth A, Kuhn M, et al. (2012) eggNOG v3.0: orthologous groups covering 1133 organisms at 41 different taxonomic ranges. Nucleic acids research 40: D284-289.

39. Krishnamurthy N, Brown DP, Kirshner D, Sjolander K (2006) PhyloFacts: an online structural phylogenomic encyclopedia for protein functional and structural classification. Genome biology 7: R83.

40. Datta RS, Meacham C, Samad B, Neyer C, Sjolander K (2009) Berkeley PHOG: PhyloFacts orthology group prediction web server. Nucleic acids research 37: W84-89.

41. Altenhoff AM, Schneider A, Gonnet GH, Dessimoz C (2011) OMA 2011: orthology inference among 1000 complete genomes. Nucleic acids research 39: D289-294.

42. Chen F, Mackey AJ, Stoeckert CJ, Jr., Roos DS (2006) OrthoMCL-DB: querying a comprehensive multi-species collection of ortholog groups. Nucleic acids research 34: D363-368.

43. Hu Z, Hung JH, Wang Y, Chang YC, Huang CL, et al. (2009) VisANT 3.5: multi-scale network visualization, analysis and inference based on the gene ontology. Nucleic acids research 37: W115-121.

44. Edgar RC (2004) MUSCLE: multiple sequence alignment with high accuracy and high throughput. Nucleic Acids Res 32: 1792-1797.

45. Felsenstein J (1989) PHYLIP--phylogeny inference package (version 3.2). Cladistics 5: 164-166.

46. Jones DT, Taylor WR, Thornton JM (1992) The rapid generation of mutation data matrices from protein sequences. Comput Appl Biosci 8: 275-282.
